# Comparative transcriptional analysis of Capsicum flower buds between a sterile flower pool and a restorer flower pool provides insight into the regulation of fertility restoration

**DOI:** 10.1186/s12864-019-6210-3

**Published:** 2019-11-11

**Authors:** Bingqiang Wei, Lanlan Wang, Paul W. Bosland, Gaoyuan Zhang, Ru Zhang

**Affiliations:** 1 College of Horticulture, Gansu Agricultural University, Lanzhou, 730070 China; 2 Vegetable Institute, Gansu Academy of Agricultural Sciences, Lanzhou, 730070 China; 3 College of Agriculture, Consumer, and Environmental Sciences, New Mexico State University, Las Cruces, 88001 USA

**Keywords:** CMS, Fertility restorer, Pepper, RNA-sequencing

## Abstract

**Background:**

Cytoplasmic male sterility (CMS) and its restoration of fertility (Rf) system is an important mechanism to produce F_1_ hybrid seeds. Understanding the interaction that controls restoration at a molecular level will benefit plant breeders. The CMS is caused by the interaction between mitochondrial and nuclear genes, with the CMS phenotype failing to produce functional anthers, pollen, or male gametes. Thus, understanding the complex processes of anther and pollen development is a prerequisite for understanding the CMS system. Currently it is accepted that the *Rf* gene in the nucleus restores the fertility of CMS, however the *Rf* gene has not been cloned. In this study, CMS line 8A and the Rf line R1, as well as a sterile pool (SP) of accessions and a restorer pool (RP) of accessions analyzed the differentially expressed genes (DEGs) between CMS and its fertility restorer using the conjunction of RNA sequencing and bulk segregation analysis.

**Results:**

A total of 2274 genes were up-regulated in R1 as compared to 8A, and 1490 genes were up-regulated in RP as compared to SP. There were 891 genes up-regulated in both restorer accessions, R1 and RP, as compared to both sterile accessions, 8A and SP. Through annotation and expression analysis of co-up-regulated expressed genes, eight genes related to fertility restoration were selected. These genes encode putative fructokinase, phosphatidylinositol 4-phosphate 5-kinase, pectate lyase, exopolygalacturonase, pectinesterase, cellulose synthase, fasciclin-like arabinogalactan protein and phosphoinositide phospholipase C. In addition, a phosphatidylinositol signaling system and an inositol phosphate metabolism related to the fertility restorer of CMS were ranked as the most likely pathway for affecting the restoration of fertility in pepper.

**Conclusions:**

Our study revealed that eight genes were related to the restoration of fertility, which provides new insight into understanding the molecular mechanism of fertility restoration of CMS in *Capsicum*.

## Background

*Capsicum* species are one of the most popular spice and vegetable crops in the world and *Capsicum annuum* is the most widely grown among the five domesticated species (*C. annuum*, *C. baccatum*, *C. chinense*, *C. frutescens*, and *C. pubescens*) [1]. Hybrid vigor is a phenomenon that is advantageous for breeders because of increased fruit yield, enhance resistance, and improve quality. However, the production of F_1_ hybrid seed needs manual emasculation that can lead to a high cost for F_1_ seed production [2]. The unique mechanism cytoplasmic male sterility (CMS) is one of the most valuable methods to utilize plant heterosis or hybrid vigor [3] because lack of pollen production removes the need for manual emasculation. Thus, breeders use the CMS/Rf system to produce hybrid seeds more economically, and the system is widely exploited for hybrid seed production of a number of crops including *Capsicum* [4–6].

Male sterility is the failure of plants to produce functional anthers, pollen, or male gametes. The CMS phenotype is maternally inherited because it is controlled by the plant’s mitochondrial genome [4]. It has been proven that CMS is caused by chimeric open reading frames (ORF) resulting from the rearrangements of the mitochondrial genome [7, 8]. These ORF may disturb the function of ATPase [9], destroy the mitochondrial membrane structure [10], and produce proteins that are cytotoxic [11], which, in turn, affect the normal development of pollen [12]. In pepper, CMS was first reported in 1958 from an Indian *Capsicum annuum* accession (PI164835) [13]. Since then, the CMS/Rf system has been used to produce F_1_ hybrid seeds of pepper [14]. Two candidate CMS genes, *orf456* and *atp6-2* loci, have since been identified [15, 16]. Another ORF, designated *orf507*, is a modified version of *orf456,* elongated through the deterioration of a stop codon. This ORF was also proven to be related to CMS and inhibit the formation of microspore in pepper [17].

The CMS phenotype can be reversed by a nuclear *Rf* gene. These genes have been found to restore fertility through several different mechanisms. The CMS transcript can be processed by a *Rf* gene at the post-transcriptional level [18]. In rice, the transcript of B-atp6-orf79 is silenced by cleavage from the restorer gene RF1A [19]. Restorer of fertility genes can also act at the translational level, as *Rfo* in radish is thought to repress the translational processing of *orf138* [20]. In maize, a putative aldehyde dehydrogenase (*rf2*) was found that may act to restore fertility through detoxification during pollen development [21].

Restorer-of-fertility genes have been found in a range of flowering plant species. They have been reported in petunia [22], radish [20, 23–26], sugar beet [27, 28], maize [21], sorghum [29], and rice [19, 30–32]. These *Rf* genes have encoded a variety of different proteins. For example, *Rf1* (*bvORF20*) encodes an OMA1-like protein in sugar beet [27, 28], *Rf2* encodes an aldehyde dehydrogenase protein in maize [21], *Rf17* gene encodes an acyl-carrier protein synthase [33] and the *Rf2* gene encodes a glycine-rich protein in rice [34], indicating the existence of a diverse set of *Rf* genes. However, most *Rf* genes are known to encode Pentatricopeptide Repeat (PPR) proteins [8, 35, 36]. In pepper, so far all predicted *Rf* genes have encoded PPRs [36, 37]. A PPR gene, *CaPPR6*, was identified as a strong *Rf* candidate based on expression pattern and characteristics of coding sequence [37]. Recently, 12 candidate PPR genes with similarity to previously reported *Rf* genes were also identified in pepper [38]. These candidate *Rf* genes provide a basis for further study of fertility restoration in pepper.

It has been suggested that fertility restorer in pepper may be controlled by two complementary genes [39]. Previous studies show that the fertility restorer of CMS in pepper is controlled by two major additive-dominant epistatic genes and an additive-dominant polygene [40], and two major QTLs and several minor QTLs [41]. It could be that the two major QTLs correspond with the two major additive-dominant genes, in which case the studies support one another. In contrast, another study indicates that one major QTL and four minor QTLs relate to fertility restoration in pepper [42]. Another phenotype of partial restoration has also been reported, in which the flower simultaneously produces functional and aborted pollen, which is thought to be controlled by a gene (*pr*) in the nucleus separate from *Rf* genes [43]. In addition, the CMS phenotype can be restored temporarily under low temperature, suggesting that temperature affects the expression of some fertility modification genes [44]. Together, these various types of fertility restoration demonstrate that CMS is complex, and currently do not have a complete understanding of the molecular mechanisms that underlie the CMS/Rf system in pepper.

The RNA-Seq method directly sequences transcripts by using high-throughput sequencing technologies, and it has considerable advantages for providing genome-wide information, detection of novel transcripts, and allele-specific expression [45]. Bulked segregate analysis (BSA) is an efficient method for the rapid identification of molecular markers for specific traits or target gene loci [46]. Combing the advantage of BSA and RNA-seq, BSA RNA-seq (BSR) can be used to analyze the differentially expressed genes (DEGs), and single nucleotide polymorphisms (SNPs) between the two genetic pools [47, 48].

In this study, BSR-seq was applied to identify DEGs related to the fertility restorer of CMS in pepper. In addition, the transcriptomes of two parent lines were sequenced. A set of candidate genes were selected that are associated with the fertility restoration in CMS in pepper based on both the BSR-seq and parental transcriptome sequencing. The results provide new insights into the study of molecular mechanisms of restorer fertility of CMS in pepper.

## Results

### Database estimation of transcript sequencing

Through the RNA-sequence of the fertility restorer line (R1), the CMS line (8A), a population of 30 fertile plants pool (RP), and a population of 30 sterile plants pool (SP), a total of 41.84 GB of aligned data were obtained. The aligned data of R1 and 8A were 8.24 GB and 6.98 GB, respectively, and that of RP and SP were 14.16 GB and 12.46 GB, respectively (Table 1). These raw data can be found in NCBI (https://www.ncbi.nlm.nih.gov/) with an accession number of SRA895207. The base ratios for quality scores of each aligned data was greater than Q30% at more than 91%, and GC content was more than 41%. This indicates that the aligned data were good for the subsequent searches.

**Table 1:** The estimation of RNA sequence data

Samples	Read Number^a^	Base Number^b^	GC Content^c^	% ≥ Q30^d^
R1	32,694,918	8,239,119,336	41.70%	91.55%
8A	27,708,115	6,982,444,980	41.82%	91.32%
RP	56,202,271	14,162,972,292	42.48%	92.06%
SP	49,443,495	12,459,760,740	42.23%	91.50%

Note: ^a^pair-end reads number in aligned data; ^b^base number in aligned data; ^c^GC content in aligned data; ^d^The percentage of bases whose quality value of aligned data is more than or equal to Q30

The aligned data had a high success rate of being mapped to a reference genome. The percentage of each aligned data being mapped to a reference genome was more than 85.50%, and the unique mapped rate was greater than 82.47% (Table 2).

**Table 2:** The comparison between sequence data and reference genomics

Samples	Total Reads	Mapped Reads^a^	Mapped Ratio^b^	Unique Mapped Reads^c^	unique Mapped Ratio^d^
R1	65,389,836	56,002,822	85.64%	54,068,310	82.69%
8A	55,416,230	47,886,185	86.41%	46,275,567	83.51%
RP	112,404,542	97,929,810	87.12%	94,135,100	83.75%
SP	98,886,990	84,549,668	85.50%	81,549,039	82.47%

Note: ^a^aligned data that were mapped to the reference genome; ^b^ percentage of aligned data being mapped to the reference genome; ^c^GC content of aligned data; ^d^ percentage of bases whose quality value of aligned data is more than or equal to Q30

### Differentially Expressed Genes (DEGs) analysis

DEGs were discovered between R1/8A and between RP/SP (Table 3). There were 3790 DEGs between R1 and 8A, in which 2274 were up-regulated and 1516 were down-regulated for expression in R1 as compared to 8A. There were also 1762 DEGs between RP and SP, in which 1490 were up-regulated and 272 were down-regulated for expression in RP as compared to SP. In an overall comparison, there were 944 co-DEGs among the above groups (R1/8A, RP/SP). Within this comparison, 891 out of 944 DEGs were commonly up-regulated in restorer accessions and the remaining 53 out of 944 DEGs were down-regulated in restorer accessions (R1 and RP) as compared to CMS accessions (8A and SP). This indicates that 891 DEGs were up-regulated not only in RP as compared to SP, but also in R1 as compared to 8A, and 53 DEGs were down-regulated not only in RP as compared to SP, but also in R1 as compared to 8A. The 891 commonly up-regulated DEGs were the subject of subsequent study.

**Table 3:** Three groups of DEGs

DEG Set	DEG number	Up-regulated DEG number	Down-regulated DEG number
R1/8A	3790	2274	1516
RP/SP	1762	1490	272
R1/RP ∩ 8A/SP	944	891	53

### Gene Ontology (GO) Annotation

**Fig. 1:**
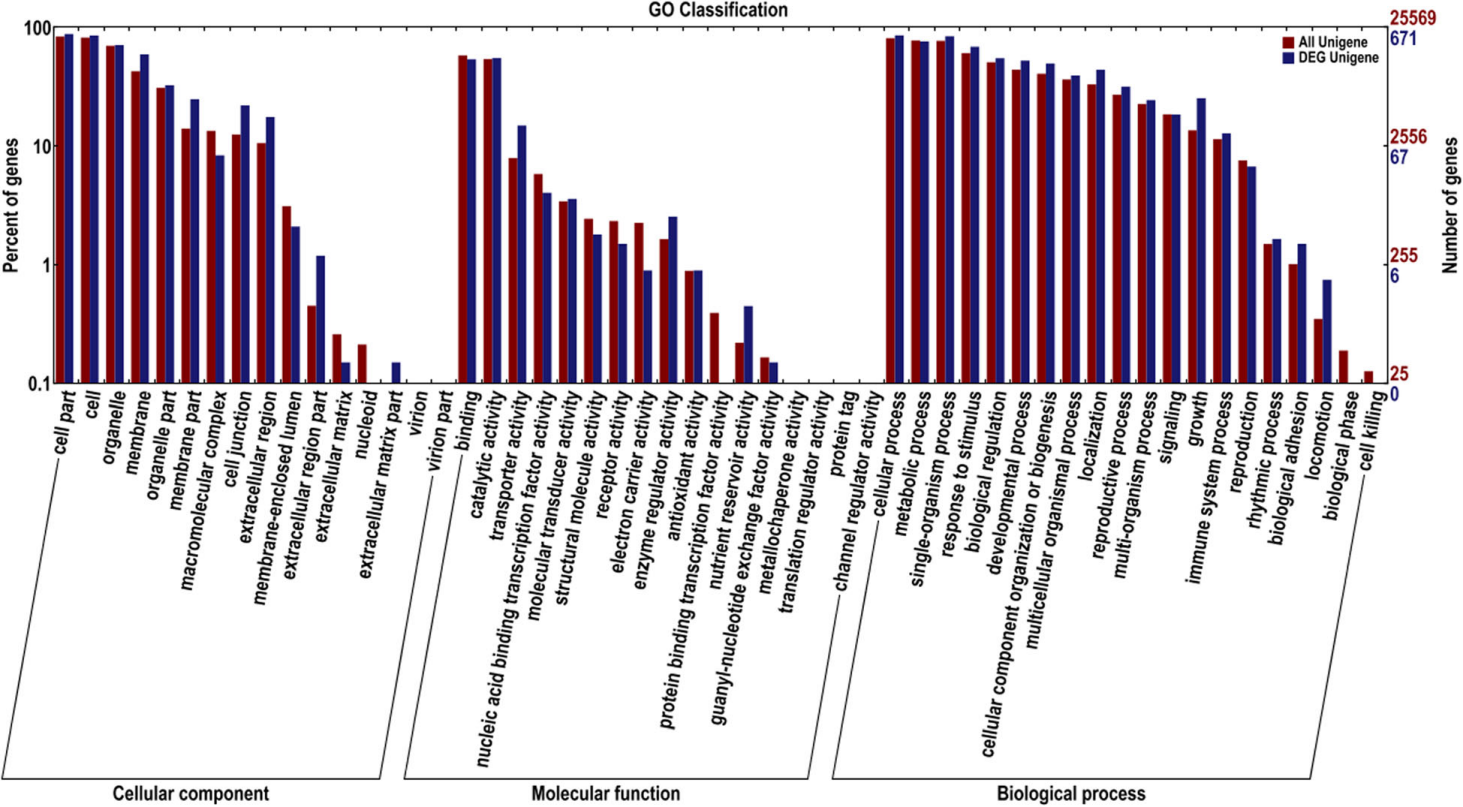
The GO functional classification of up-regulated genes. Red bars represent all expressed genes, and blue bars represent DEGs

The 891 common up-regulated DEGs in restorer accessions were assigned to three main categories, cellular component, molecular function, and biological process. These three categories were composed of 53 functional groups using GO assignment (Fig.1).

In the cellular component category, the majority functional groups were cell part, cell, organelle, and membrane, including 589, 573, 475, and 397 genes, respectively. The most significant GO node that is enriched to the DEGs is external side of plasma membrane (GO:0009897), composed of Capana01g001287, Capana01g00392, Capana06g000150, Capana01g004065 and Capana09g002397 totaling 5 up-regulated DEGs (Table 4). The next were GO:0009535, GO:0005615, GO:0030173, GO:0005618, GO:0048046 and GO:0016021, including 4, 5, 6, 91, 60 and 97 up-regulated DEGs, respectively (Table 4).

**Table 4:** The enrichment results for the cellular component DEGs by topGO

GO ID^a^	Term^b^	Annotated^c^	Significant^d^	Expected^e^	KS^f^
GO:0009897	external side of plasma membrane	131	5	3.63	3.20E-07
GO:0009535	chloroplast thylakoid membrane	715	4	19.83	5.90E-05
GO:0009522	photosystem I	89	0	2.47	0.00023
GO:0005615	extracellular space	95	5	2.63	0.00035
GO:0030173	integral to Golgi membrane	24	6	0.67	0.00047
GO:0005618	cell wall	2279	91	63.21	0.00064
GO:0048046	apoplast	1336	60	37.06	0.00085
GO:0009523	photosystem II	104	0	2.88	0.00133
GO:0030076	light-harvesting complex	43	0	1.19	0.00134
GO:0016021	integral to membrane	2151	97	59.66	0.00153

Note: ^a^GO term ID; ^b^GO function; ^c^all genes annotated the function; ^d^DEGs annotated the function; ^e^Expected value of the DEGs annotated the function; ^f^Statistical significance of enrichment nodes, the smaller the KS value, the more significant enrichment

In the molecular function category, the dominant function types were catalytic activity and binding with 369 and 360 genes, respectively. The most significant GO node that is enriched to the DEGs is serine-type endopeptidase activity (GO:0004252), composed of Capana04g000159, Capana06g000150, Capana09g002397, Capana05g000136, Capana11g002184 and Capana01g001287 totaling 6 up-regulated DEGs (Table 5). The next GO nodes were protein kinase activity (GO:0004672), transmembrane receptor protein kinase activity (GO:0019199), 8-hydroxyquercitin 8-O-methyltransferase activity (GO:0030761) and isoflavone 4'-O-methyltransferase activity (GO:0030746), including 56, 4, 1 and 1 up-regulated DEGs, respectively (Table 5).

**Table 5:** The enrichment results of molecular function DEGs by topGO

GO ID^a^	Term^b^	Annotated^c^	Significant^d^	Expected^e^	KS^f^
GO:0004252	serine-type endopeptidase activity	175	6	4.71	1.20E-13
GO:0004672	protein kinase activity	2053	56	55.27	1.50E-08
GO:0019199	transmembrane receptor protein kinase activity	203	4	5.46	5.30E-08
GO:0036202	ent-cassa-12,15-diene 11-hydroxylase activity	32	0	0.86	4.90E-06
GO:0010327	acetyl CoA:(Z)-3-hexen-1-ol acetyltransferase activity	14	0	0.38	8.70E-06
GO:0048038	quinone binding	53	0	1.43	2.20E-05
GO:0030761	8-hydroxyquercitin 8-O-methyltransferase activity	44	1	1.18	2.40E-05
GO:0030746	isoflavone 4′-O-methyltransferase activity	44	1	1.18	2.40E-05
GO:0047203	13-hydroxylupinine O-tigloyltransferase activity	15	0	0.4	3.10E-05
GO:0033793	aureusidin synthase activity	10	0	0.27	4.30E-05

Note: ^a^GO term ID; ^b^GO function; ^c^all genes annotated the function; ^d^DEGs annotated the function; ^e^Expected value of the DEGs annotated the function; ^f^Statistical significance of enrichment nodes, the smaller the KS value, the more significant enrichment

In the biological process category, the dominant terms were metabolic process, response to stimulus, biological regulation, developmental process and cellular component organization or biogenesis including 574, 564, 511, 460, 368, 351, and 332 genes, respectively. The most significant GO node that is enriched to the DEGs is mucilage extrusion from seed coat (GO:0080001), composed of Capana04g000159, Capana05g000136, apana09g002397, Capana01g001287 and Capana06g000150 totaling five up-regulated DEGs (Table 6). The next nodes were GO:0016045, GO:0006898, GO:0010204, GO:0048359, GO:0010359, GO:0010102 and GO:0052544, including 9, 6, 1, 10, 7, 9 and 8 up-regulated DEGs, respectively (Table 6).

**Table 6:** The enrichment results of the biological process DEGs by topGO

GO ID^a^	Term^b^	Annotated^c^	Significant^d^	Expected^e^	KS^f^
GO:0080001	mucilage extrusion from seed coat	109	5	2.96	1.30E-15
GO:0002764	immune response-regulating signaling pathway	135	1	3.66	3.80E-13
GO:0016045	detection of bacterium	271	9	7.36	2.50E-12
GO:0006898	receptor-mediated endocytosis	139	6	3.77	3.30E-10
GO:0010204	defense response signaling pathway, resistance gene-independent	157	1	4.26	3.40E-09
GO:0048359	mucilage metabolic process involved seed coat development	205	10	5.56	7.90E-09
GO:0010359	regulation of anion channel activity	339	7	9.2	8.60E-09
GO:0010102	lateral root morphogenesis	322	9	8.74	5.00E-08
GO:0009864	induced systemic resistance, jasmonic acid mediated signaling pathway	107	0	2.9	1.20E-06

Note: ^a^GO term ID; ^b^GO function; ^c^all genes annotated the function; ^d^DEGs annotated the function; ^e^**Expected value** of the DEGs annotated the function; ^f^Statistical significance of enrichment nodes, The smaller the KS value, the more significant enrichment

### Kyoto Encyclopedia of Genes and Genomes (KEGG ) Metabolic Pathway of DEGs

**Fig. 2:**
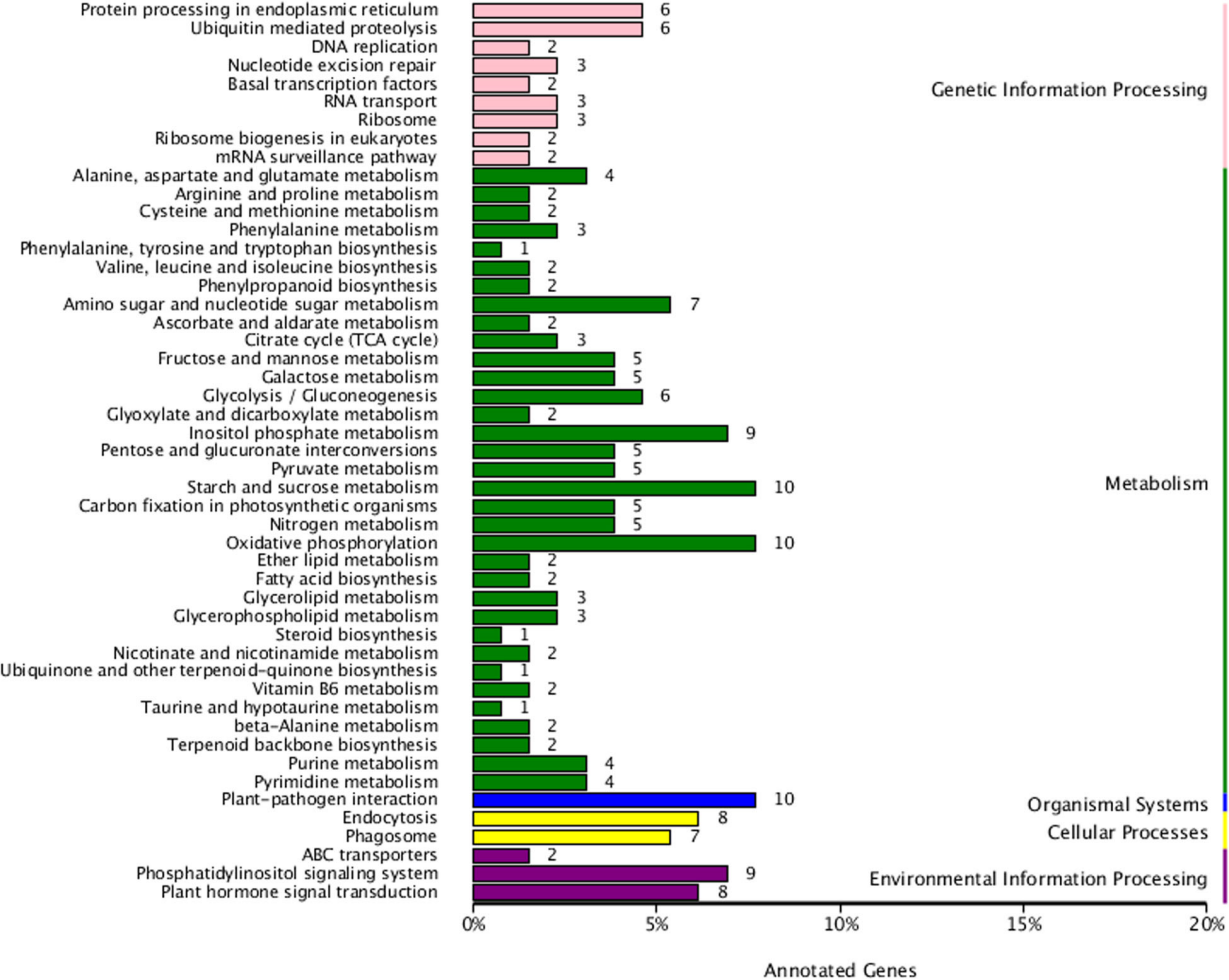
Statistic analysis of up-regulated differentially expressed genes in KEGG pathways

**Fig. 3:**
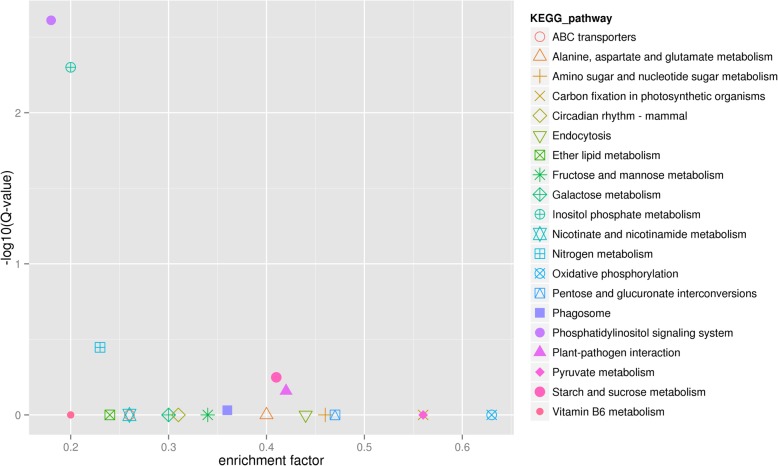
Scatter plot of KEGG pathway enrichment for up-regulated DEGs. Each small shape in the figure represents a KEGG path, and the path names are shown in the legend on the right. The horizontal coordinate is the enrichment factor, the smaller the enrichment factor, the more significant the enrichment level of DEGs in the pathway. The ordinate is -log_10_ (Q value), the larger the ordinate, the more reliable the enrichment of DEGs in the pathway

The up-regulated shared DEGs were annotated to 49 KEGG metabolic pathways in five categories including genetic information processing, metabolism, organismal systems, cellular processes, and environmental information processing (Fig. 2). The metabolic pathways composed of the most up-regulated DEGs were starch and sucrose metabolism, oxidative phosphorylation, and plant-pathogen interaction. The next metabolic pathways composed of nine up-regulated DEGs were inositol phosphate metabolism, and the phosphatidylinositol signaling system (Fig. 2). KEGG pathways that may be involved in fertility recovery or pollen development include energy metabolism, carbohydrate metabolism, protein and amino acid metabolism, lipid metabolism, substance absorption and transport, and signal transduction (Additional File 1: Table S1). Interestingly, two metabolic pathways, phosphatidylinositol signaling system and inositol phosphate metabolism, were enriched as the most reliable pathway for enrichment significance (Fig. 3). Within these two metabolic pathways most genes were the same, with eight out of nine genes being shared between the two pathways.

**Fig. 4:**
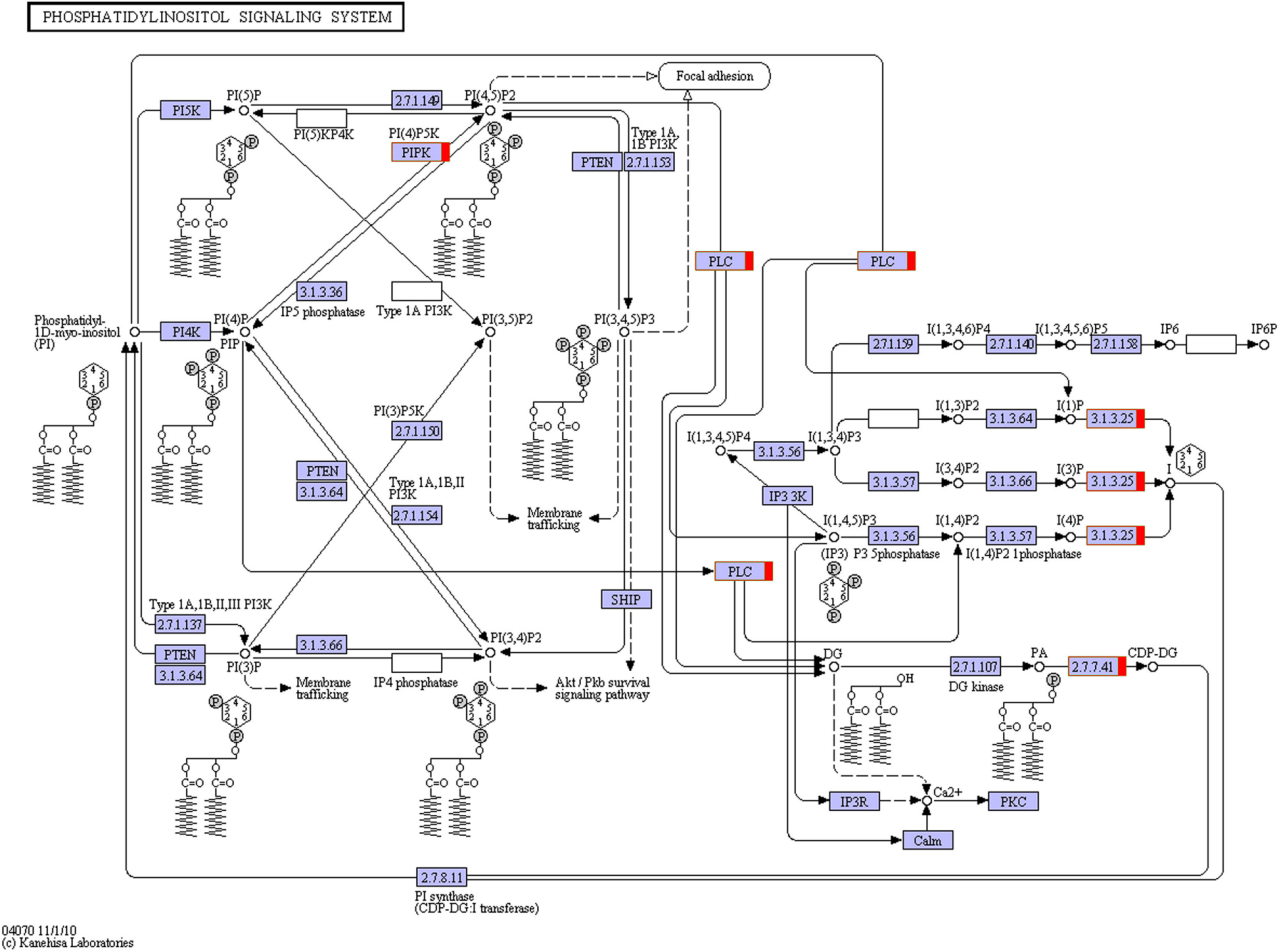
The gene node participating in phosphatidylinositol signaling system. The red square frame means up-regulated expressed genes in the node

In the phosphatidylinositol signaling system (ko04070), scored as the most enriched system, nine genes were up-regulated (Fig. 4). Among these nine genes, five (Capana00g002844; Capana00g004424; Capana10g001436; Capana10g002170; Capana10g002470) encode phosphatidylinositol-4-phosphate 5-kinase (PI(4)P5K) and catalyzes the phosphatidylinositol-4-phosphate (PI(4)P) to phosphatidylinositol-4,5-biphosphate (PI(4,5)P_2_). Meanwhile, two genes (Capana03g002795 and Capana06g002131) encode phospholipase C (PLC), which could hydrolyze phosphatidylinositol (PI), PI(4)P or PI(4,5)P_2_ to generate double messenger molecules inositol triphosphate (IP_3_) and diacylglycerol (DG). Two genes (Capana05g000173 and Capana07g002321) encode inositol phosphate phosphatase and phosphatidate cytidylyltransferase, respectively, which could participate in the reduction of IP_3_ and DG to PI.

### Related Genes Selection and expression

According to conjoined analysis of the gene FPKM (fragments per kilobase of exon per million fragments mapped) value, difference multiple, qRT-PCR value, functional annotation and metabolic pathway classification, eight genes, related to fertility restoration of CMS in *Capsicum* were selected. The eight genes were Capana00g002348, Capana00g004424, Capana02g000930, Capana00g003267, Capana01g002849, Capana05g002270, Capana01g004065 and Capana06g002131, and they encode fructose kinase, phosphatidylinositol phosphokinase, pectin lyase, extragalacturonase, pectin esterase, cellulose synthase, and bundle arabinogalactan protein, respectively (Table 7). All listed genes are key enzymes and proteins in anther and pollen development.

**Table 7:** The selected genes related to the restorer-of-fertility and their expression and function annotation

Gene ID	FPKM				log_2_FC		Nr_annotation
	8A	R_1_	SP	RP	R_1_/8A	RP/SP	
Capana00g002348	0.086539	0.264261	1.76E-06	0.611852	8.59032	9.6092	putative fructokinase-5-like [*Solanum tuberosum*]
Capana00g004424	0	1.56466	0	2.64384	7.27376	7.7515	phosphatidylinositol 4-phosphate 5-kinase 4-like [*Solanum tuberosum*]
Capana02g000930	0.280479	150.854	1.74488	322.03	8.97438	7.50312	pectate lyase-like [*Nicotiana tomentosiformis*]
Capana00g003267	0.112148	1.39687	0	3.16586	10.2657	6.57239	expolygalacturonase clone GBGE184-like [*Nicotiana tomentosiformis*]
Capana01g002849	7.51E-05	1.2932	6.12E-06	21.9292	5.49358	11.4473	pectinesterase4-like [*Solanum lycopersicum*]
Capana05g002270	0	2.81676	2.06E-05	10.1792	8.25692	9.93493	cellulose synthase-like protein D1-like [*Solanum tuberosum*]
Capana01g004065	0.148665	55.2292	0.128079	158.337	8.39073	9.87305	fasciclin-likearabinogalactan protein 3 [*Solanum lycopersicum*]
Capana06g002131	5.21E-05	1.04156	0.203871	0.65287	4.64828	4.27352	PLC

**Fig. 5:**
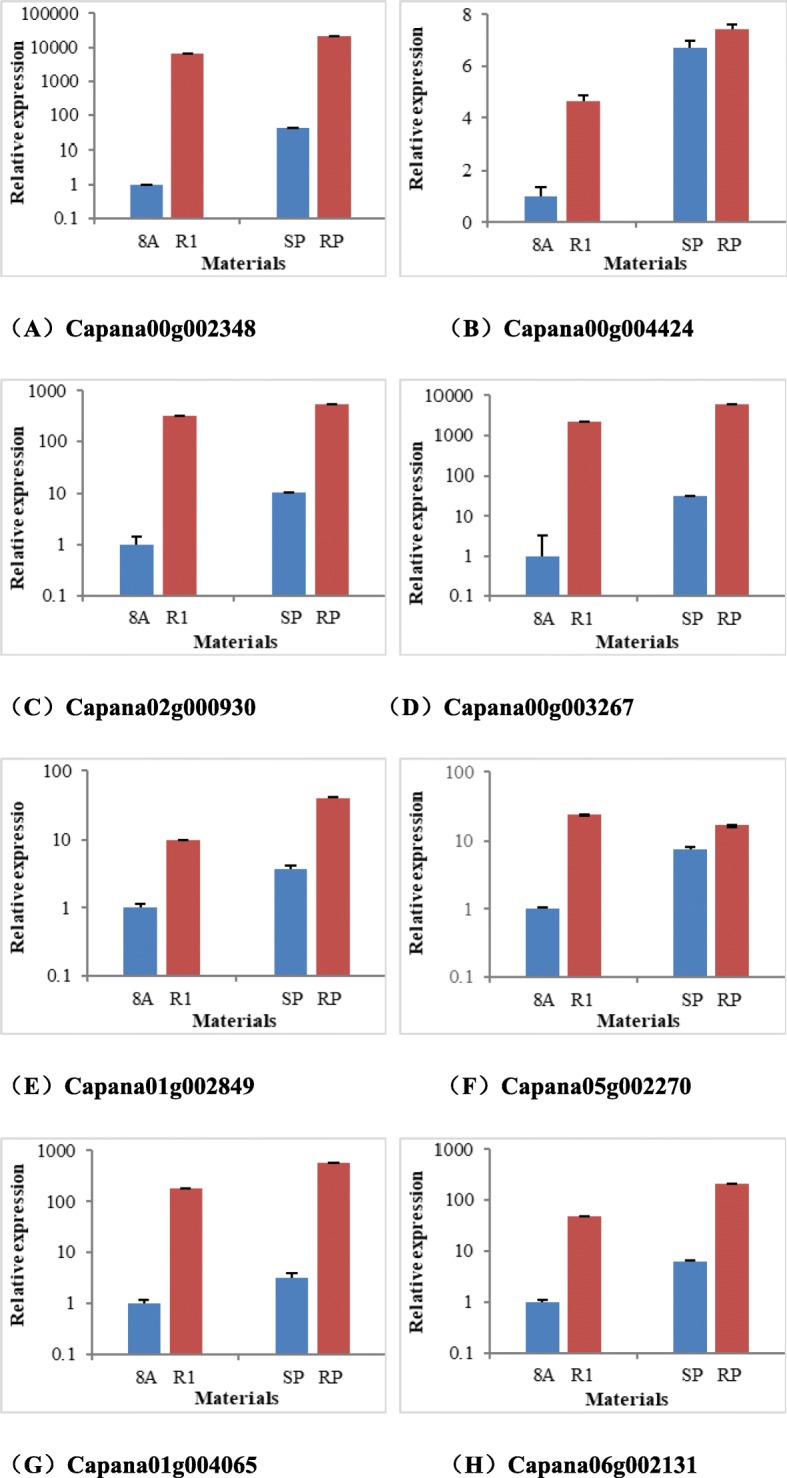
The qRT-PCR validation of eight genes related to fertility restorer between 8A and R1, as well as SP and RP. (**a**) The relative expression of Capana00g002348 in four accessions. (**b**) The relative expression of Capana00g004424 in four accessions. (**c**) The relative expression of Capana02g000930 in four accessions. (**d**) The relative expression of Capana00g003267 in four accessions. (**e**) The relative expression of Capana01g002849 in four accessions. (**f**) The relative expression of Capana05g002270 in four accessions. (**g**) The relative expression of Capana01g004065 in four accessions. (**h**) The relative expression of Capana06g002131 in four accessions

According to RNA-sequencing, these genes were dramatically up-regulated in the restorer parent (R1) and restorer pool (RP). The majority of these selected genes related to fertility restoration showed very little expression in the CMS 8A and SP plants, and one selected gene, Capana00g004424, had no detected expression in CMS line 8A and SP (Table 7). The lack of expression of these genes was validated by qRT-PCR between 8A and R1, as well as SP and RP. The qRT-PCR results indicated that these genes were up-regulated in both R1 and RP, as compared to 8A and SP, which was completely consistent with the sequencing results (Fig. 5).

**Fig. 6:**
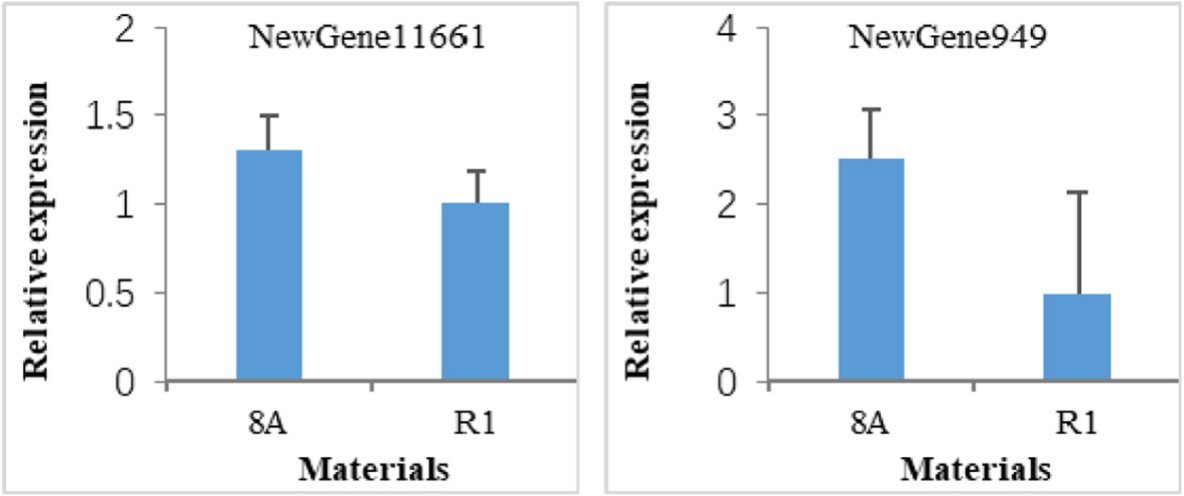
The qRT-PCR validation of two obviously down-regulated genes between 8A and R1

The relative expression of two obviously down-regulated genes (NewGene11661 and NewGene949) both in fertile accessions R1 and RP as compared to sterile accessions 8A and SP were validated by qRT-PCR. The results indicated that two genes were also down-regulated in R1 compared to 8A (Fig. 6).

**Fig. 7:**
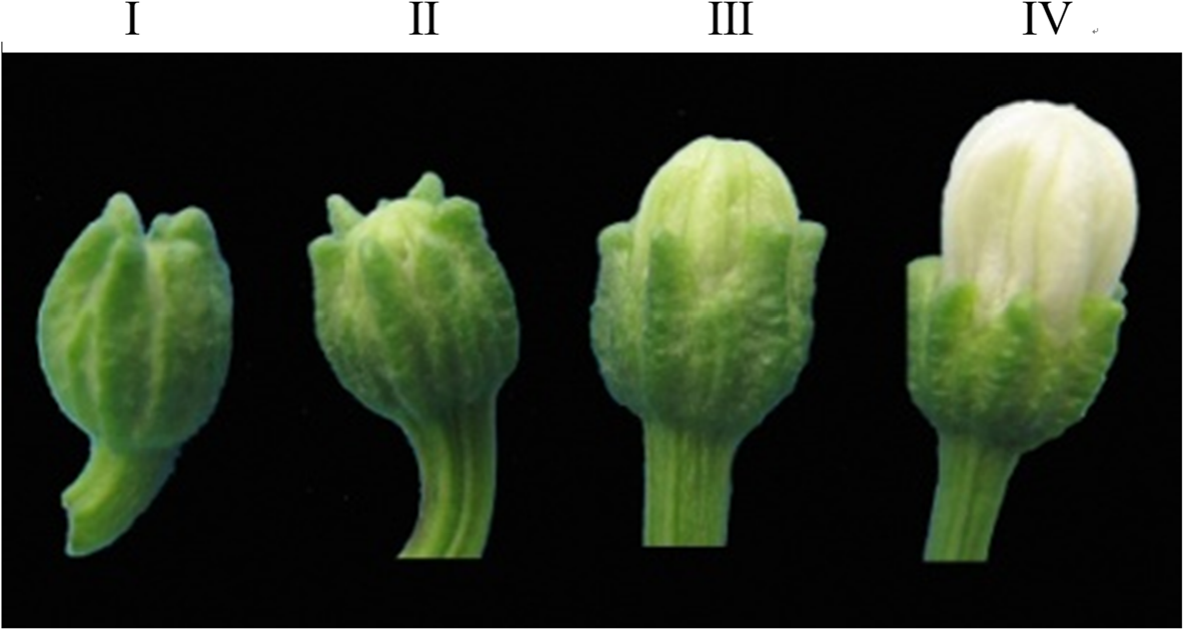
The four developmental stages of flower buds used for qRT-PCR analysis

**Fig. 8:**
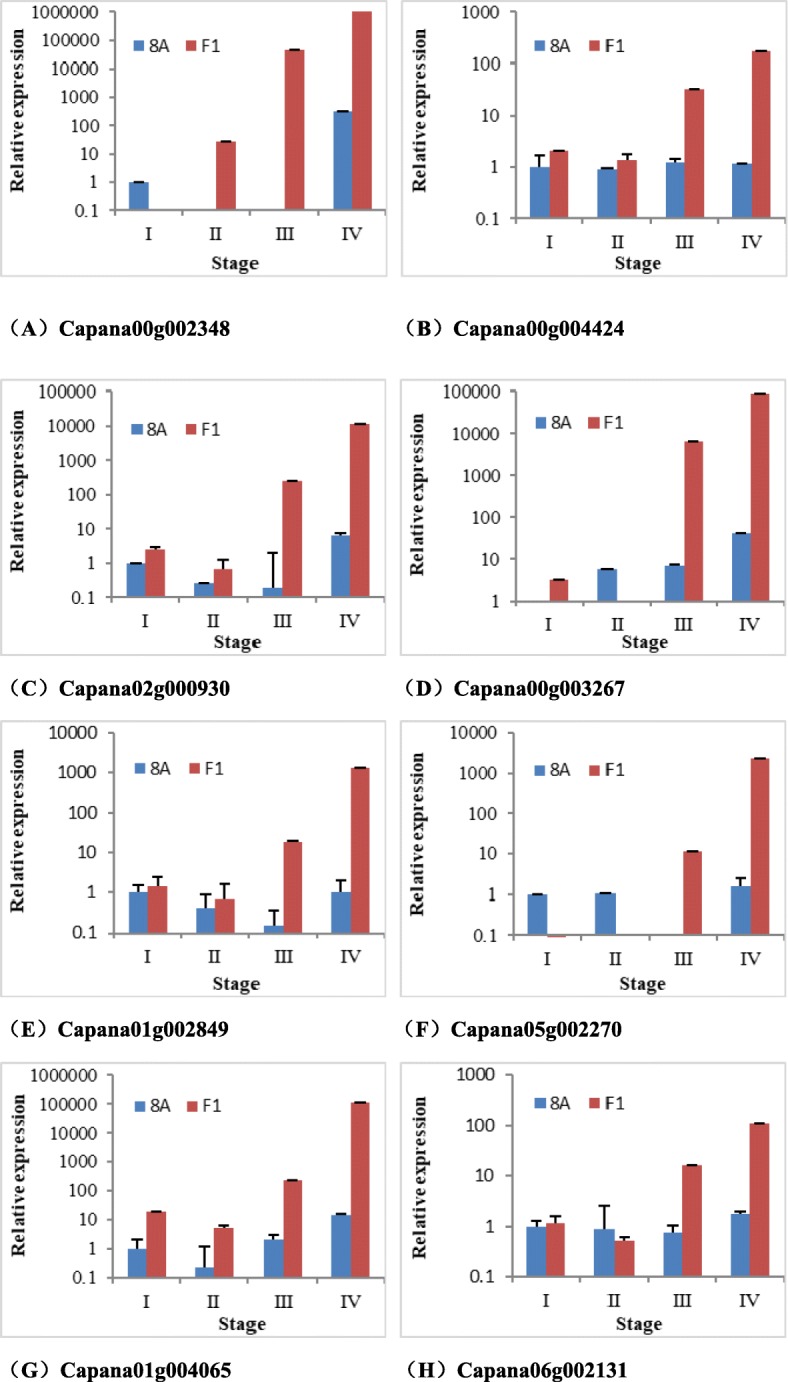
The expression of genes related to fertility restorer in different developmental stage of buds between 8A and F_1_. (**a**) The relative expression of Capana00g002348. (**b**) The relative expression of Capana00g004424. (**c**) The relative expression of Capana02g000930. (**d**) The relative expression of Capana00g003267 in four accessions. (**e**) The relative expression of Capana01g002849. (**f**) The relative expression of Capana05g002270. (**g**) The relative expression of Capana01g004065. (**h**) The relative expression of Capana06g002131

Tissue from four different developmental stage buds tested the relative expression, and the different stages are shown in Fig. 7. The qRT-PCR results show that the expression of these genes had a lower and relatively stable level among four developmental stages of flower buds in 8A. However, in the F_1_ generation, the relative expression varied among the different developmental stages. In the F_1_ generation, as the flower buds developed, the relative expression had a dramatic increase. At stage III, the expression increased rapidly until it peaked at stage IV (Fig. 8).

**Fig. 9:**
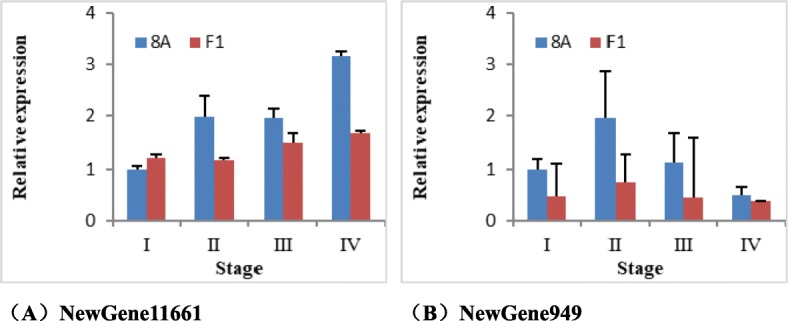
The relative expression of two down-regulated genes in different developmental stage of buds between 8A and F_1_. (**a**) The relative expression of NewGene11661. (**b**) The relative expression of NewGene949

In addition, the relative expression of two down-regulated genes were analyzed in four developmental stages of the buds between 8A and F_1_. The relative expression of NewGene11661 improved with the development of flower buds in two accessions, and the expression in every stage of 8A were higher than in R1 (Fig. 9). Unfortunately, although the relative expression of NewGene949 was higher in every stage in 8A than in R1, there was not agreement with the expected tendency (Fig. 9).

## Discussion

Male sterility and the fertility restorer system are an extremely complex biological process involving substance and energy metabolism, signal transduction pathway, substance transportation, pollen wall morphogenesis, tapetum formation and programmed cell death (PCD), and a series of related gene expression regulation processes.

Anthers are the strongest energy reservoir in the flower organs, and have a very active metabolism during development. A large number of sugars are transported to the anther [49], which can regulate the expression of genes as both a substrate of carbohydrate and a signal molecule in anthers [50]. In this study, one gene encoding fructokinase had an up-regulated expressed value of log_2_FC more than 8.5 times in R1/8A as well as RP/SP in a sucrose and starch metabolism pathway, which indicated that fructokinase is one of the key genes that regulates the fertility restorer of CMS in pepper.

Generally, pectin and callose in pollen mother cell (PMC) are degraded absolutely, otherwise the microspore cannot be separated from the tetrad [51, 52]. The pectin methylesterification, is degraded by de-methylated esterification first by methylesterases (PMEs), then the combined action of PME, pectin lyases (PLs), and polygalacturonases (PGs) [53, 54]. In Arabidopsis, QRT1 gene encodes a PME, QRR2, and QRT3 encoding an external PG and an endonuclear PG, respectively. The loss of function of QRT1 didn’t reduce the level of pectin methylesterification. If the level of the pectin methylesterification is higher, it cannot be degraded by PG [55]. In this study, three genes encoding PME, PL and exo-PG were selected. What is more, these genes had up-regulated expression and the log_2_FC were as high as 11.4473, 10.2657, and 8.97438, respectively. The results presented are in agreement with Hamid et al [56] that showed that eight pectin lyase-like superfamily protein coding genes and five pectin methylesterase genes were up-regulated in fertile plants as compared to Cytoplasmic Genic Male Sterility (CGMS) in cotton. In addition, a fasciclin-like protein gene was also up-regulated in restorer materials in this study. This result was similar to the reports of Hamid et al. [56], in which three cytoskeleton organization genes were up-regulated in fertile lines in cotton. These up-regulated genes may have a positive role in the degradation of pectin and the normal release of microspores.

It is well known that celluloses and hemicelluloses are the important components of pollen intine. Cellulose composed of a class of β-1,4-glucan molecular long chain plays an important role in cell wall toughness and strength. The deposition of cellulose on intine is mainly carried out by cellulose synthase complexes (CSCs) that is located on the cell membrane [57, 58]. In *Arabidopsis*, the deposition of cellulose in intine and extine was not well-distributed in the mutant of CESA1 gene and CESA3 gene, which resulted in abnormal pollen development. Fortunately, we also choose one gene encoding cellulose synthase that is up-regulated dramatically in restorer accessions.

It is commonly thought that the phosphatidylinositol signal system is one of the important signal systems for plant seed germination, growth and reproduction, senescence and response to environmental factors [59]. Pollen development undergoes a series of complex cell division and differentiation processes, which involve the dynamic changes of many cell components and internal subcellular components, including vacuole and cytoskeleton [60]. Many components of phosphatidylinositol signaling system participate in the vacuolar diversification during pollen development and vesicle transport in pollen tube growth. In this study, eight genes were up-regulated in phosphatidylinositol signal system. There are five genes that encode PI(4)P5K, which can catalyze PI(4)P to PI(4,5)P_2_. Previous studies have shown that PI(4)P is very important for pollen and stigma affinity, and PI(4,5)P_2_ plays an important role in vesicle transport and cell skeleton rearrangement [61]. PI(4)P5K is a very important enzyme in the development of the anther, it would lead to an abnormal morphology of the pollen tube if PI(4)P5K was lost, thus inhibiting the germination of pollen and the growth of the pollen tube [62–65]. In *Arabidopsis*, PIP5K1 and PIP5K2 are important for vacuole biogenesis and early pollen development, pollen grains from flowers of the pip5k1+/−pip5k2+/−mutants show defects in vacuoles and exine wall formation [65]. In addition, two genes encode PLC, and PLC is the most important in phosphatidylinositol signal system, which could hydrolyze PI, PI(4)P and PI(4,5)P_2,_ to double as the messenger molecules inositol trisphosphate (IP_3_) and diacylglycerol (DG). It is also known that there is a calcium dependent PLC activity in pollen tubes, and PLC and IP_3_ are involved in the germination and growth of pollen tubes [66]. PLC can monitor the growth of the pollen tube not only by adjusting the content of PI(4,5)P_2_, but also by regulating the concentration of Ca^2+^ and membrane secretion.

The development of pollen walls plays a key role in the development of pollen, and it leads to male sterility in plants if the development of the pollen outer wall is blocked. Sporopollenin is the main substance that constitutes the outer wall of pollen, and the sporopollenins are mainly composed of polymerized phenols and long-chain fatty acid derivatives, which are difficult to decompose and can effectively protect pollen from degradation from the outside. There is also a thick oil-bearing layer outside of the pollen grain, including hydrophobic lipids and proteins, composed of long-chain fatty acid derivatives [67, 68]. Previous studies showed that sporopollenin biosynthesis is closely associated with fatty acid metabolism [69, 70]. Therefore, lipid accumulation plays an important role in pollen morphogenesis. In this study, some up-regulated genes related to lipid metabolism pathway were discovered, and these metabolic genes may be involved in the synthesis of lipids in the tapetum, which are then transported to the pollen wall surface (Additional file 1: Table S1). It had also been reported in cabbage that in the protein interaction network around CYP704B1, more than 1/3 of the protein species were involved in fatty acid metabolism, and all of them were significantly reduced in 83121A [71].

Vesicle transport and lipid carrier transport are the two main modes of sporopollen transportation. Adenosine triphosphate binding cassette (ABC) is a protein family in which ABC members were involved in the transmembrane transport of substances. In *Arabidopsis thaliana*, more than 120 ABC proteins have been identified. They are involved in the transport of ions, hormones, secondary metabolites, lipids, and proteins. The ABCG26/GBC27 belongs to the G subfamily of ABC protein and is expressed in microspore and tapetum, which may be involved in the transport of sporopollen [72, 73]. In this study, two up-regulated ABC transport genes were discovered that may be involved in the transportation of sporopollen (Additional file 1: Table S1).

In addition, the synthesis of fatty acid needs ATP and acetyl-CoA that come from cell respiration and energy metabolism. Plant energy metabolism mainly includes the tricarboxylic acid cycle, glycolysis, pentose phosphate pathway, and the electron transport and oxidative phosphorylation. This energy metabolism provides ATP and acetyl-CoA for the synthesis of fatty acids. Previous studies have shown that the impairment of respiratory chain enzymes and enzyme complexes can lead to CMS [74, 75]. Similar to the results in eggplant [76], this study found many up-regulated genes in the restorer line to participate in energy pathways, such as oxidative phosphorylation, glycolysis, and citrate cycle (Additional file 1: Table S1).

The relative expression of NewGene11661 was down-regulated in the F_1_ while up-regulated in 8A, and the expression increased with the development of flower buds. However, it did not improve more dramatically than other candidate genes. NewGene11661 was annotated as UDP-glycosyltransferase gene. The UDP glycosyltransferase could catalyze conjugation of lipophilic chemicals with sugar donated by UDP-glycoside, generating water-soluble products that can be easily excreted and resulting in the detoxification and elimination of their substrate [77, 78]. However, the roles of this enzyme in the development pollen or CMS have been rarely reported and needs further study.

## Conclusion

By RNA sequencing, in this study 891 DEGs were up-regulated and 53 DEGs were down-regulated in restorer accessions as compared to the CMS accessions. The 891 up-regulated DEGs in restorer accessions were assigned to three main functional categories: cellular component, molecular function, and biological process, all of which were composed of 53 functional groups using GO assignment. The up-regulated DEGs were annotated to 49 KEGG metabolic pathways in five categories including genetic information processing, metabolism, organismal systems, cellular processes, and environmental information processing. As in Fig. 3, two metabolic pathways, phosphatidylinositol signaling system and inositol phosphate metabolism, were statistically the most likely pathways for affecting fertility restoration. Finally, eight DEGs were selected that encoded fructose kinase, phosphatidylinositol phosphokinase, pectin lyase, extragalacturonase, pectin esterase, cellulose synthase, and bundle arabinogalactan protein. The qRT-PCR results showed that the expression of these genes had a lower and relatively stable level between four developmental stages of the flower bud in 8A. But in the F_1_ generation, with the development of flower buds, the relative expression had a tendency to significant increase, especially at the stage III, where the expression increased rapidly and peaked at stage IV. The tendency to significantly increase the eight candidate genes supports RNA differential expression and are likely to be involved in pepper fertility restoration.

## Materials and Methods

### Plant sample collection and preparation

**Fig. 10:**
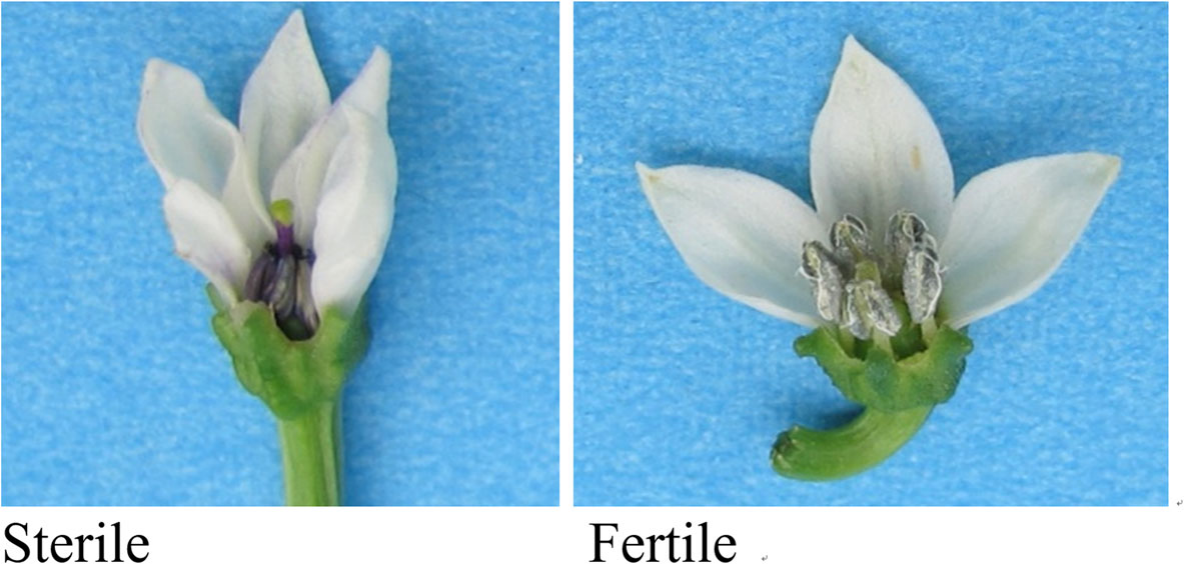
The flower pictures of CMS line 8A and fertility restorer line R1

The CMS 8A line was completely sterile, and the fertility restorer line R1 completely restored the fertility of 8A (Fig. 10). The F_2_ population from the self-pollination of the F_1_ individuals showed segregation for fertility. The restore fertility pool (RP) was constructed from 30 polar fertile plants and the sterile pool (SP) was constructed from 30 polar sterile plants. The flower buds of 8A, R1, SP, and RP at the tetrad stage were used for total RNA extraction. Four different developmental stages of flower buds of 8A and F_1_ analyzed the relative expression of fertility restoration related genes.

### RNA extraction and library construction and RNA Sequencing

Total RNA was extracted separately from the flower buds of 8A, R1, SP, and RP according to the instructions of Trizol Reagent (Life technologies, California, USA). The RNA purity was checked using the NanoPhotometer® spectrophotometer (IMPLEN, CA, USA). The RNA concentration was measured using Qubit® RNA Assay Kit in Qubit®2.0 Flurometer (Life Technologies, CA, USA). The RNA integrity was assessed using the RNA Nano 6000 Assay Kit of the Agilent Bioanalyzer 2100 system (Agilent Technologies, CA, USA). Then the mRNA was enriched using magnetic beads with Oligo (dT) for reverse transcription. The cDNA library was constructed following the manufacturer’s instructions for NEBNext Ultra RNA Library Prep Kit for Illumina (NEB, E7530). Finally, the cDNA libraries of the four flower buds from 8A, R1, SP, and RP have been sequenced on a flow cell using the Illumina HiSeq™ 2500 sequencing platform.

### Transcriptome analysis using reference genome-based reads mapping

Low quality reads, such as adaptor sequences, unknown nucleotides greater than 5%, or low quality score (Q-value)≤ 20 bases, were removed by Perl script. The aligned reads that were filtered from the raw reads were mapped to the Pepper Genome Database (release 2.0) [79] using TopHat2 software [80]. The aligned records in BAM/SAM format examined to remove potential duplicate molecules. Gene expression levels were estimated using FPKM values by the Cufflinks software [81]. Identification of new genes was based on transcript discovery with Cufflinks, and then filtering the genes encoding for a short peptide chain (less than 50 amino acid residues). By comparing the raw gene model and the result of transcriptome assembly, gene structure was optimized. SpliceGrapher integrated splice graphs generated from Cufflinks output and read alignments to produce comprehensive alternative splicing predictions [82].

### Identification of DEGs

To evaluate DEGs among lines 8A, R1, SP, and RP, the EBSeq [83] was employed with screening criteria FC ≥ 2 / log_2_FC ≥ 1 and False Discovery Rate (FDR) < 0.01. The differential multiple / FC represents the ratio of the expression between the two groups. By adjusting the difference between P-values, the FDR is obtained. Because the differential expression analysis of transcriptome sequencing is an independent statistical hypothesis test for a large number of gene expression values, there is a false positive problem. To address this, in the process of differential expression analysis, the accepted Benjamini-Hochberg correction method corrected the significant *P*-value obtained from the original hypothesis test, and finally FDR was used as the key index for screening DEGs.

### GO classification and enrichment analysis

Perl script plotted GO functional classification for the unigenes with a GO term hit to view the distribution of gene functions. GO enrichment analysis of the DEGs was implemented by the GOseq R packages based Wallenius non-central hyper-geometric distribution [84], which can adjust for gene length bias in DEGs.

### KEGG pathway and enrichment analysis

KEGG is a database resource for understanding high-level functions and utilities of the biological system, such as the cell, the organism, and the ecosystem, from molecular-level information, especially large-scale molecular datasets generated by genome sequencing and other high-throughput experimental technologies (http://www.genome.jp/kegg/) [85]. This study used KOBAS software to test the statistical enrichment of differential expression genes in KEGG pathways [86].

### Screening of fertility restoration related genes

Genes related to fertility were selected according to comprehensive estimation of gene log_2_FC value and gene function. Firstly, genes that were dramatically up-regulated (log_2_FC ≥ 7) in restorer accessions as compared to sterile accessions were selected. Genes were selected on the basis of having FPKM values higher in restorer accessions and having FPKM values close to zero in sterile accessions (Table 7). Secondly, this group was filtered according to function, choosing genes playing an important role in the normal development of pollen as found in previous studies, or are involved mostly in the important or enriching pathways in this study to further evaluate.

### Expression analysis of fertility restoration related genes

Extraction of RNA was performed under the conditions described above. The first strand cDNA synthesis was carried out using the 1st strand cDNA synthesis kit (Revert Aid Premium Reverse Transcriptase) (Thermo Scientific, EP0733). A total of eight fertility restorer related genes and two down-regulated genes were tested for their relative expression and the *CaActin* (GenBank Accession: GQ339766.1) gene was the internal control (Table 8). The total volume of each reaction is 20 μL. Using SG Fast qPCR Master Mix (High Rox) (2×), qRT-PCR was carried out (BBI, B639273). The qRT-PCR was performed on an ABI Stepone plus Real-Time PCR System (Applied Biosystems, USA) with the following cycling parameters: 95°C for 3 m, followed by 45 cycles of: 95 °C for 7 s, 57°C for 10 s, 72°C for 15 s. The relative expression was calculated by using the 2^−ΔΔCt^ method described by Livak and Schmittgen [87]. All reactions were performed with at least three replicates.

**Table 8:** The test genes and their primers for qRT-PCR

Gene	Forward Primer		Reverse Primer		length
	Sequence	Tm	Sequence	Tm	
Capana00g002348	AGGGTCCAAAATGGGCTGT	59.0	CTCCCCAAAACATACGACGA	58.3	190
Capana00g004424	CAGGTGAATGGAAGAATGGTG	57.7	ACATAAAAGCTCCCATCAGTCC	57.8	139
Capana02g000930	TGCAAAGCTACAAAACCATCG	59.2	ATCACCTTCATCCCCAGTCCT	59.6	191
Capana00g003267	GTGGTGGTACTCTAAACGGACAA	58.7	TGAATCTGGTGCTGTAATCTTGAG	59.1	215
Capana01g002849	TATGGCGATGGATGATTGC	57.2	GGTAGGCGTAGACCTTTGAAAG	58	149
Capana05g002270	CCCTTGAGGAACTATGGCGTA	59.5	CTTCATCATCACCAGCAGACTTT	58.7	150
Capana01g004065	TGTTGTAGGCTCTGTGCGTG	57.7	GGCTGAGATAGGTGCCATTG	57.9	133
Capana06g002131	TCAGGGCTAATGGTGGTTGT	57.7	TTCTTTACTTCATCAGCAGGGA	57.1	238
NewGene11661	GCCCATCGCTGTTCTTATTG	58.3	TGAAGACGGGCGTGAGTGT	59.2	116
NewGene949	TTTGAGGTGGGGTGGTTGT	57.9	AGTGGTTGCCATGAAGGTTG	58.1	166
CaActin	TGCCTGATGGACAAGTTATTACC	58.8	TGAGCACAATGTTACCGTAGAGG	59.7	174

## Supplementary information

**Additional file 1: Table S1.** The KEGG Pathway related to the pepper restoration of fertility or pollen development.

## Data Availability

All data generated and analyses done within this study are included in this published article and its supplementary information files. The raw data can be found in NCBI (https://www.ncbi.nlm.nih.gov/) with an accession number of SRA895207.

## Abbreviations

ABC: triphosphate binding cassette
BSA: Bulked segregate analysis
BSR: BSA RNA-seq
CGMS: Cytoplasmic Genic Male Sterility
CMS: Cytoplasmic male sterility
CSCs: Cellulose synthase complex
DEG: Differentially expressed gene
DG: Diacylglycerol
FC: Fold Change
FDR: Discovery Rate
FPKM: Fragments per kilobase of exon per million fragments mapped
GO: Gene Ontology
IP_3_: Inositol triphosphate
KEGG: Kyoto Encyclopedia of Genes and Genomes
ORF: Open reading frames
PCD: Programmed cell death
PG: Polygalacturonase
PI (4) P: Phosphatidylinositol-4-phosphate
PI (4) P5K: Phosphatidylinositol-4-phosphate 5-kinase
PI (4,5) P_2_: phosphatidylinositol-4,5-biphosphate
PI: Phosphatidylinositol
PL: Pectin lyase
PLC: Phospholipase C
PMC: Pollen mother cell
PME: Methylesterase
PPR: Pentatricopeptide Repeat
qRT-PCR: quantitative real-time polymerase chain reaction
QTL: Quantitative trait loci
Rf: Fertile restorer / restoration of fertility
RP: Restorer pool
SP: Sterile pool
