# Supplementary material for: Comparative transcriptional analysis of Capsicum flower buds between a sterile flower pool and a restorer flower pool provides insight into the regulation of fertility restoration
Source: BMC Genomics. 2019 Nov 11;20:837. doi: 10.1186/s12864-019-6210-3 (PMC6849218; doi:10.1186/s12864-019-6210-3)
Supplement: Supplementary file 1 — Additional file 1: Table S1. The KEGG Pathway related to the pepper restoration of fertility or pollen development. [file 12864_2019_6210_MOESM1_ESM.doc]

**Table S1** The KEGG Pathway related to the pepper restoration of fertility or pollen development

| ClassiFication | Pathway | ko_ID | Unigene | Gene ID | K_ID |
| --- | --- | --- | --- | --- | --- |
| energy metabolism | Citrate cycle (TCA cycle) | ko00020 | 3 | Capana01g003447；Capana02g002466；Capana12g000832 | K00025；K00030；K01610 |
|  | Glycolysis/ Gluconeogenesis | ko00010 | 6 | Capana02g002913；Capana04g001331；Capana06g000334；Capana09g002320；Capana11g001371；Capana12g000832 | K00844；K03841；K01568；K00873；K00134；K01610 |
|  | Pentose phosphate pathway | ko00030 | 1 | Capana04g001331 | K03841 |
|  | Oxidative phosphorylation | ko00190 | 10 | Capana01g000018;Capana01g004491；Capana02g001311；Capana02g002840；Capana03g000501；Capana07g000268；Capana07g001645；Capana07g001646；Capana09g000062；Chilli_newGene_14744 | K01507；K01507；K02152；K02133；K01535；K01535；K03885；K03885；K01535；K02150 |
| carbohydrate metabolism | Starch and sucrose metabolism | ko00500 | 10 | Capana00g001535；Capana00g002348；Capana00g003267；Capana00g003785；Capana00g004616；Capana01g000522；Capana02g002158；Capana02g002913；Capana03g004067；Capana10g001560 | K01193；K00847；K01213；K00963；K00975；K01193；K01051；K00844；K00696；K01193 |
|  | Fructose and mannose metabolism | ko00051 | 5 | Capana00g002348；Capana02g002913；Capana04g001331；Capana07g001325；Chilli_newGene_11699 | K00847；K00844；K03841；K00895；K01711 |
|  | Galactose metabolism | ko00052 | 5 | Capana00g001535；Capana00g003785；Capana01g000522；Capana02g002913；Capana10g001560 | K01193；K00963；K01193；K00844；K01193 |
|  | Amino sugar and nucleotide sugar metabolism | ko00520 | 7 | Capana00g002348；Capana00g003452；Capana00g003785；Capana00g004616；Capana02g002913；Capana09g000440；Chilli_newGene_11699 | K00847；K12450；K00963；K00975；K00844；K12448；K01711 |
|  | N-Glycan biosynthesis | ko00510 | 1 | Capana04g000140 | K01228 |
|  | Pentose and glucuronate interconversions | ko00040 | 5 | Capana00g003267；Capana00g003785；Capana02g000930；Capana02g002158；Capana11g001625 | K01213；K00963；K01728；K01051；K01728 |
| Protein and amino acid metabolism | Ubiquitin mediated proteolysis | ko04120 | 6 | Capana04g000436；Capana06g002866；Capana06g002871；Capana06g003066；Capana10g001973；Capana12g002483 | K10610；K10579；K10579；K03347；K10591；K03094 |
|  | beta-Alanine metabolism | ko00410 | 2 | Capana00g003786；Capana08g002465 | K01580；K05605 |
|  | Phenylalanine metabolism | ko00360 | 3 | Capana01g004380；Capana11g000163；Capana12g000857 | K00430；K11188；K00815 |
|  | Tyrosine metabolism | ko00350 | 1 | Capana12g000857 | K00815 |
|  | Cysteine and methionine metabolism | ko00270 | 2 | Capana12g000261；Capana12g000857 | K01251；K00815 |
|  | Phenylalanine, tyrosine and tryptophan biosynthesis | ko00400 | 1 | Capana12g000857 | K00815 |
|  | Valine, leucine and isoleucine biosynthesis | ko00290 | 2 | Capana02g001264；Capana10g001960 | K01869；K01754 |
|  | Valine, leucine and isoleucine degradation | ko00280 | 1 | Capana08g002465 | K05605 |
|  | Tryptophan metabolism | ko00380 | 1 | Capana08g000749 | K11816 |
|  | Arginine and proline metabolism | ko00330 | 2 | Capana05g001715；Capana05g001995 | K01915；K00261 |
|  | Glycine, serine and threonine metabolism | ko00260 | 1 | Capana10g001960 | K01754 |
|  | Alanine, aspartate and glutamate metabolism | ko00250 | 4 | Capana00g003786；Capana05g001715；Capana05g001995；Capana06g002516 | K01580；K01915；K00261；K01953 |
| lipid metabolism | Glycerolipid metabolism | ko00561 | 3 | Capana06g001189；Capana09g000045；Capana12g001160 | K00679；K03715；K13519 |
|  | Ether lipid metabolism | ko00565 | 2 | Capana01g003818；Capana12g001160 | K01115；K13519 |
|  | Fatty acid metabolism | ko00071 | 1 | Capana01g004475 | K01897 |
|  | Fatty acid biosynthesis | ko00061 | 2 | Capana03g001691；Capana09g000140 | K10781；K09458 |
|  | Biosynthesis of unsaturated fatty acids | ko01040 | 1 | Capana06g002523 | K10257 |
|  | Glycerophospholipid metabolism | ko00564 | 3 | Capana01g003818；Capana07g002321；Capana12g001160 | K01115；K00981；K13519 |
| Vitamin metabolism | Ascorbate and aldarate metabolism | ko00053 | 2 | Capana05g000173；Capana12g000346 | K10047；K00469 |
|  | Vitamin B6 metabolism | ko00750 | 2 | Capana00g003162；Capana00g003163 | K13248；K13248 |
| Material absorption and transport | Phagosome | ko04145 | 7 | Capana02g001311；Capana05g000386；Capana06g000422；Capana06g001088；Capana11g000775；Capana12g000592；Chilli_newGene_14744 | K02152；K05692；K05692；K08054；K05692；K07375；K02150 |
|  | Endocytosis | ko04144 | 8 | Capana00g002844；Capana00g004424；Capana01g003818；Capana10g000060；Capana10g001436；Capana10g001973；Capana10g002170；Capana10g002470 | K00889；K00889；K01115；K12486；K00889；K10591；K00889；K00889 |
|  | ABC transporters | ko02010 | 2 | Capana02g001595；Chilli_newGene_17818 | K05658；K05658 |
|  | RNA transport | ko03013 | 3 | Capana01g000745；Capana06g000349；Capana06g001200 | K03260；K00784；K03257 |
|  | SNARE interactions in vesicular transport | ko04130 | 1 | Capana00g004541 | K08490 |
| Signal transduction | Plant hormone signal transduction | ko04075 | 8 | Capana02g000676；Capana02g003021；Capana03g000009；Capana03g002801；Capana07g001573；Capana08g002278；Capana08g002647；Chilli_newGene_12772 | K14487；K14487；K14488；K14497；K14487；K14487；K14488；K14487 |
|  | Phosphatidylinositol signaling system | ko04070 | 9 | Capana00g002844；Capana00g004424；Capana03g002795；Capana05g000173；Capana06g002131；Capana07g002321；Capana10g001436；Capana10g002170；Capana10g002470 | K00889；K00889；K05857；K10047；K05857；K00981；K00889；K00889；K00889 |
|  | Inositol phosphate metabolism | ko00562 | 9 | Capana00g002844；Capana00g004424；Capana03g002795；Capana05g000173；Capana06g002131；Capana10g001436；Capana10g002170；Capana10g002470；Capana12g000346 | K00889；K00889；K05857；K10047；K05857；K00889；K00889；K00889；K00469 |
| Others | Basal transcription factors | ko03022 | 2 | Capana00g003330；Capana10g002352 | K03139；K03124 |
|  | Terpenoid backbone biosynthesis | ko00900 | 2 | Capana05g002501；Capana11g000901 | K01823；K14066 |
|  | Pyruvate metabolism | ko00620 | 5 | Capana01g003447;Capana03g001354;Capana07g002124;Capana09g002320;Capana12g000832 | K00025+K01638+K01595+K00873+K01610 |
